# Feasibility of a cluster randomised trial on the effect of trauma life support training: a pilot study in India

**DOI:** 10.1136/bmjopen-2025-099020

**Published:** 2025-12-10

**Authors:** Martin Gerdin Wärnberg, Debojit Basak, Johanna Berg, Shamita Chatterjee, Li Felländer-Tsai, Geeta Ghag, Catherine Juillard, Monty Khajanchi, Tamal Khan, Anurag Mishra, Vipul Versi Nandu, Nobhojit Roy, Rajdeep Singh, Kapil Dev Soni, Lovisa Strömmer

**Affiliations:** 1 Department of Global Public Health, Karolinska Institutet, Stockholm, Sweden; 2 Perioperative Medicine and Intensive Care, Karolinska University Hospital, Stockholm, Sweden; 3 Seth Sukhlal Karnani Memorial Hospital, Kolkata, West Bengal, India; 4 Institute of Postgraduate Medical Education and Research, Kolkata, West Bengal, India; 5 Department of Internal and Emergency Medicine, Skånes universitetssjukhus Malmö, Malmo, Sweden; 6 Department of Surgery, Seth Sukhlal Karnani Memorial Hospital, Kolkata, West Bengal, India; 7 Department of Clinical Science Intervention and Technology (CLINTEC), Karolinska Institutet, Stockholm, Sweden; 8 Department of Reconstructive Orthopedics, Karolinska University Hospital, Stockholm, Sweden; 9 Department of Surgery, HBT Medical College and Dr RN Cooper Municipal General Hospital, Mumbai, Maharashtra, India; 10 Division of General Surgery, Department of Surgery, David Geffen School of Medicine, University of California Los Angeles, Los Angeles, California, USA; 11 Department of Surgery, Seth GS Medical College and KEM Hospital, Mumbai, Maharashtra, India; 12 All India Institute of Medical Sciences, New Delhi, India; 13 Department of Surgery, Maulana Azad Medical College, New Delhi, India; 14 Program for Global Surgery and Trauma, The George Institute for Global Health India, New Delhi, India; 15 Critical Care, All India Institute of Medical Sciences, New Delhi, India

**Keywords:** Trauma, TRAUMA MANAGEMENT, Emergency Departments

## Abstract

**Objective:**

To assess the feasibility of conducting a cluster randomised controlled trial comparing the effects of Advanced Trauma Life Support (ATLS) and Primary Trauma Care (PTC) with standard care on patient outcomes.

**Design:**

This was a pilot pragmatic three-armed parallel, cluster randomised, controlled trial conducted between April 2022 and February 2023. Patients were followed up for 30 days.

**Setting:**

Tertiary care hospitals across metropolitan areas in India.

**Participants:**

Adult trauma patients and residents managing these patients were included.

**Interventions:**

ATLS or PTC training was provided for residents in the intervention arms.

**Main outcomes and measures:**

The outcomes were the consent rate, loss to follow-up rate, missing data rates, differences in the distribution between observed data and data extracted from medical records, and the resident pass rate.

**Results:**

Two hospitals were randomised to the ATLS arm, two to the PTC arm and three to the standard care arm. We included 376 patients and 22 residents. The percentage of patients who consented to follow-up was 77% and the percentage of residents who consented to receive training was 100%. The loss to follow-up rate was 14%. The pass rate was 100%. Overall, the amount of missing data for key variables was low. The data collected through observations were similar to data extracted from medical records, but there were more missing values in the extracted data.

**Conclusions:**

Conducting a full-scale cluster randomised controlled trial comparing the effects of ATLS, PTC and standard care on patient outcomes appears feasible, especially if such a trial would use data and outcomes available in medical records.

**Trial registration number:**

NCT05417243.

**STRENGTHS AND LIMITATIONS OF THIS STUDY**

- Prospective data collection with direct observations made by dedicated research officers.
- A lack of a priori defined success criteria and thresholds for feasibility outcomes.
- The use of sealed envelopes potentially compromised allocation concealment.
- Heterogeneity of the participating centres may affect the study estimates and introduce bias.

## Introduction

 Trauma, defined as a clinical entity composed of physical injury and the associated response of the body, causes 4.3 million deaths every year.^1^ Several trauma life support training programmes have been developed to improve the early management of patients in hospitals by providing a structured framework for assessment and treatment.^2^,^7^

Advanced Trauma Life Support (ATLS) and Primary Trauma Care (PTC) are the most established trauma life support training programmes.^8 9^ Both programmes improve provider knowledge and skills,^2 4^ and while most observational studies associate them with reduced mortality,^10^,^20^ some report associations with increased mortality.^21 22^

There are no controlled trials of the effect of ATLS or PTC on patient outcomes, and no trials evaluating any trauma life support training programme in a general trauma population.^2^,^7^ However, a recent cluster randomised trial found that the rural trauma team development course reduced mortality in patients with motorcycle injuries.^23^

Systematic reviews call for controlled trials of ATLS and PTC,^2^,^4^ but large scale cluster randomised trials can be complex. We, therefore, conducted a pilot study with the aim to assess the feasibility of a cluster randomised controlled trial comparing the effects of ATLS and PTC with standard care on outcomes in adult trauma patients.

## Methods

### Protocol deviations

Protocol deviations are mentioned where relevant in this manuscript, but a list of all deviations is also included as online supplemental Material S1 for completeness.

### Trial design

We piloted a three-armed cluster randomised controlled trial.^24^ The trial included a standard care arm and two intervention arms, ATLS and PTC training. We planned to collect data for 4 months in all three arms, first during a 1-month observation phase, followed by a 3-month intervention phase (or continued observation in the standard care arm). The actual data collection period varied across clusters depending on the timing of the training, to ensure a minimum of 3 months of data collection in the intervention clusters post-training. We included a 1-month observation phase to evaluate the feasibility of comparing patient outcomes both as absolute differences between the intervention phases, and as differences in changes from baseline. In the published protocol, we also aimed to estimate probable effect sizes and other parameters needed for sample size calculations for a full-scale trial.^24^ However, we revised this aim in light of current guidance on the conduct and reporting of pilot trials.^25^

### Study setting

We conducted this pilot study in seven tertiary hospitals across metropolitan areas in India, where neither ATLS, PTC nor any other established trauma life support training programme is routinely taught. Details about each cluster are provided in online supplemental Table S1. We initially intended to include six hospitals as clusters, but added a seventh hospital when management expressed interest, as we had the budget to accommodate the request. These seven hospitals represented a convenience sample that fulfilled the inclusion criteria and had existing connections to the research team.

### Eligibility criteria for cluster and participants

#### Clusters

We defined a cluster as a tertiary care hospital in metropolitan areas in India that admits more than 400 adult patients with trauma annually, and has operating theatres, X-ray, CT and ultrasound facilities, and blood bank available around the clock. In each cluster, we trained one or more units of physicians providing trauma care in the emergency department. To be eligible, units could have no more than 25% of their physicians with previous training in either ATLS, PTC or similar training programmes. Residents who had received training in the last 5 years were considered trained. The 25% threshold was determined through consensus within the research team, to balance feasibility and the risk of contamination. The principal investigator at each hospital selected the units for training. We randomised at the hospital level to avoid contamination between the intervention and standard care arms.

#### Residents

We trained resident doctors undertaking specialty training in surgery or emergency medicine who managed trauma patients in the emergency department and were expected to remain in the participating hospitals for at least 1 year after training. Consent was obtained from the residents in each intervention arm before ATLS or PTC training. In the published protocol, we stated that only surgical residents would be trained. However, in some of the participating hospitals, emergency medicine residents led the initial resuscitation and management of trauma patients, and we therefore included them in the training.

#### Patients

We included persons aged 15 years or older who presented to the emergency department at participating hospitals with a history of trauma when a designated unit was on duty. A history of trauma was defined as having any of the external causes of morbidity and mortality listed in block V01–Y36, chapter 20 of the International Classification of Disease version 10 (ICD-10) codebook as the reason for presentation.

### Standard care

Standard care varies across hospitals in India, but most surgical and emergency medicine departments in India organise their physicians into units. These units include both faculty members and residents, who are assigned a specific day of the week to work in the emergency department. Trauma patients are initially assessed by residents in these units, who also resuscitate patients, perform interventions and refer patients for imaging or other investigations. Compared with settings that adopt a trauma team approach, nurses and other healthcare professionals are involved in initial management only to a limited extent. We did not collect data on how standard care varied between the participating hospitals.

### Intervention

In each intervention arm, residents from one or two units were trained in either ATLS or PTC at the beginning of the 3-month intervention phase. For the purpose of this pilot study, our target was to train at least 75% of residents in each unit. Faculty members were not trained because they are typically not directly involved in the initial management of trauma patients. ATLS training was conducted at an ATLS-certified training centre in Mumbai and PTC training was conducted in New Delhi. Both trainings were conducted according to their respective standard curriculum,^8 9^ and we did not modify or adapt the delivery or content of these programmes during this pilot study.

The provider courses of both programmes take place across two days and focus on the assessment, resuscitation and stabilisation of trauma patients, with adaptations for different patient populations. Teaching is based on case discussions and skill stations. There are several important differences between the two programmes. The ATLS course focuses more on interhospital patient transfers and includes a greater emphasis on the trauma team.^8^ In contrast, the PTC course focuses on trauma care in the low-resource setting.^9^ The ATLS programme is run by the American College of Surgeons and requires a participant fee, whereas the PTC programme is run by the UK-based PTC Foundation and is provided free of charge.

### Feasibility outcomes

Our feasibility outcomes were as follows:

- Consent rates of patients and residents: This was defined as the percentage of patients or residents who consented to be included, out of the total number of eligible patients or residents.
- Loss to follow-up rate: This applied only to patients and was defined as the percentage of patients among all included patients who did not complete the 30-day follow-up.
- Missing data rate: This applied to each outcome and variable and was defined as the percentage of missing values.
- Differences in distributions between directly observed data and data extracted from medical records: Distribution refers to summary statistics and directly observed data refers to data collected by project officers while observing the delivery of care. This outcome applied to all variables that could be reasonably expected to be present in the medical records. To reduce workload, these data were extracted from a convenience sample of patients only.
- Pass rate: This applied only to residents in the intervention arms and was defined as the percentage of residents who passed the training programme, among all residents who received training.

We did not prespecify criteria to determine whether to proceed with a full-scale trial.

### Sample size

We aimed to include at least two clusters per arm to avoid basing conclusions on single centres. We also aimed to train at least two units per intervention cluster to evaluate the logistics of sending residents for training. We did not conduct a formal power calculation for this pilot, as the primary purpose of the study was to assess the feasibility of the trial logistics and research methods. We anticipated variation in the number of patients included per cluster depending on hospital patient volume.

### Participant timeline and inclusion

#### Patients

Incoming patients were screened for eligibility and consented, if they were conscious and able to provide consent. For unconscious patients, consent was provided by a patient representative. These patients reaffirmed this proxy consent, on regaining consciousness. Patients who did not regain consciousness remained included based on their representative’s consent. We followed up patients at 24 hours and at 30 days after arrival at the emergency department. The follow-up period for each patient was therefore 1 month.

#### Residents

Participating units were screened for eligibility once the hospitals confirmed participation. All residents in these units were approached for consent to training if their hospital was randomised to one of the intervention arms. The protocol stated that residents would be approached for consent before randomisation, but this proved not to be feasible. Instead, we asked residents for consent after the hospitals were randomised but before training. Training took place approximately 1 month after study initiation in that hospital. We initially planned to use simple random sampling to select the units to be trained, but for pragmatic reasons, the decision on which units to train was left to the site principal investigator. The number of residents trained in each intervention cluster varied based on the unit size.

### Allocation and blinding

We used simple randomisation implemented using sealed envelopes to allocate sites to the trial arms. It was not possible to blind investigators, residents or patients to the intervention. Data analysts were not blinded.

### Data collection

The planned data collection period was 4 months. However, the actual period varied across clusters depending on the timing of the training, to ensure a minimum of 3 months of data collection after the training in the intervention clusters. Research officers collected data on all patients who presented on the days and shifts when participating residents were assigned to trauma care. The research officers observed care, interviewed residents and patients and extracted data from hospital records. Admitted patients were followed up for complications and other in-hospital outcome measures. Patients who were not admitted or who were discharged before the end of the study were followed up by telephone for mortality and quality of life.

### Variables

Research officers collected data on demographics, vital signs, management details including imaging and surgery, and details of any injury sustained. All injuries were coded according to the ICD-10. Based on these codes, we calculated the Injury Severity Score (ISS) using the R package icdpicr.^26^ The ISS is a widely used measure of injury severity and ranges from 0 to 75, with a cut-off score of 16 often used to define major trauma and 75 representing unsurvivable trauma. We also collected data on potential outcomes for the full-scale trial, including 30-day and in-hospital mortality, complications and health-related quality of life. We assessed health-related quality of life using the EuroQol-5 Dimension-3 Level (EQ-5D-3L). We did not calculate an EQ-5D-3L index score because no Indian value set is currently available.^27^ We also attempted to collect data on the cause of death. A list of variables is available in online supplemental Table S2.

### Patient and public involvement

We conducted community consultations with patients, their caregivers, patient groups and resident doctors to inform the selection of outcome measures and implementation of the full-scale trial. Results of these consultations are published separately.^28^ We initially planned to distribute periodic surveys to residents and follow them up 30 days after training, but this was later changed to end-of-study interviews to allow for richer data (not published).

### Data monitoring

Weekly online meetings were held to monitor study progress and data collection. One interim analysis was conducted approximately halfway through the study, and it was decided to complete the study, as residents and patients were consenting to be included in the study and key variables including mortality could be collected. A formal data monitoring committee was not used.

### Statistical methods

All analyses were conducted using R V.4.5.0 (2025-04-11) statistical software.^29^ Feasibility outcomes and other data were analysed using descriptive statistics, and no formal hypothesis testing was performed. Initially, we planned to analyse feasibility outcomes at both the overall and individual cluster levels, but the sample sizes in individual clusters were too small to generate meaningful results. Quantitative variables are summarised as medians and IQRs. Qualitative variables are presented as absolute numbers and percentages. Additional analyses performed according to the original protocol are available as additional online material.^30^

## Results

**Figure 1:**
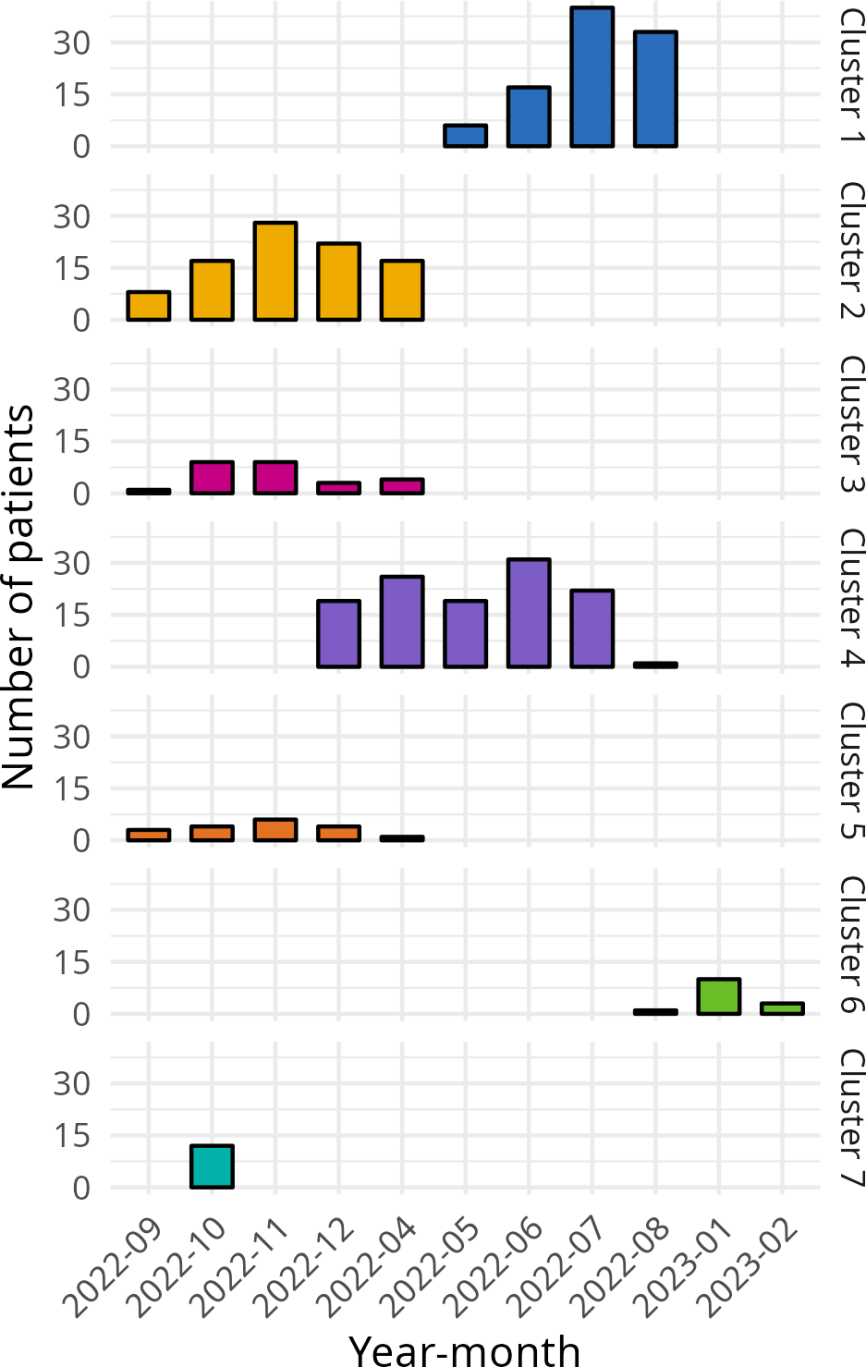
Number of patients included per cluster per month. Owing to an error in the data uploading process, data were available for only 1 and 3 months in two clusters, respectively.

We included 376 trauma patients from 7 clusters between April 2022 and February 2023. The data collection period and the number of patients included per month per cluster are shown in figure 1. Owing to an error in the data uploading process, data were available only for 1 and 3 months in two clusters, respectively. The standard care arm included 202 patients, the ATLS arm included 44 patients and the PTC arm included 130 patients. A total of 22 residents were trained: 7 in ATLS and 15 in PTC.

**Figure 2:**
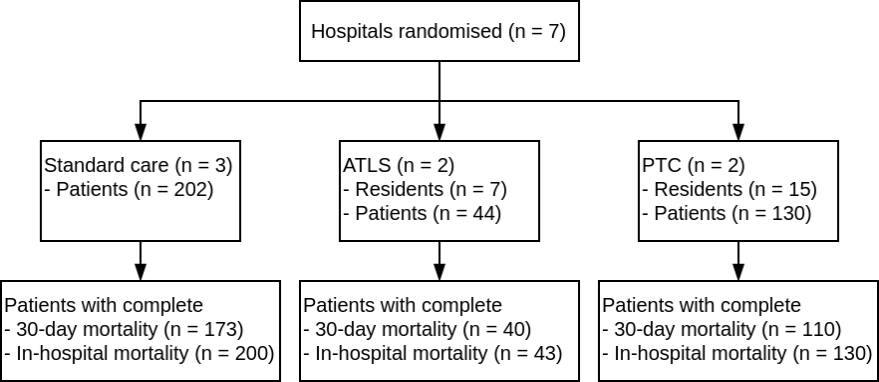
Study flow diagram. ATLS, Advanced Trauma Life Support; PTC, Primary Trauma Care.

The study flow diagram is shown in figure 2, and patient sample characteristics across trial arms are shown in table 1. Extended patient sample characteristics are shown in online supplemental Table S2. Overall, 86 (23%) patients were females, the median (IQR) age was 33 (24, 46) years and the median ISS (IQR) was 4 (1, 8). These prognostic factors differed between the trial arms. A total of 32 (10%) patients died within 30 days of arrival at the emergency department, and 29 (8%) patients died in the hospital.

**Table 1:** Patient sample characteristics

Characteristic	Standard care N=202	ATLS N=44	PTC N=130	Overall N=376
Age, years, median (IQR)	35 (25, 47)	40 (30, 57)	30 (22, 38)	33 (24, 46)
Elderly (age ≥65 years), n (%)	15 (7%)	6 (14%)	5 (4%)	26 (7%)
Sex, n (%)				
Male	160 (79%)	33 (75%)	97 (75%)	290 (77%)
Female	42 (21%)	11 (25%)	33 (25%)	86 (23%)
Dominating injury type, n (%)				
Penetrating	13 (6%)	3 (7%)	1 (1%)	17 (5%)
Blunt	189 (94%)	41 (93%)	129 (99%)	359 (95%)
Blunt multisystem trauma, n (%)	2 (1%)	2 (5%)	6 (5%)	10 (3%)
Severe traumatic brain injury, n (%)	10 (5%)	1 (2%)	5 (4%)	16 (4%)
Missing	1	0	0	1
Shock (SBP ≤90 mm Hg), n (%)	4 (2%)	2 (5%)	4 (3%)	10 (3%)
Missing	7	3	4	14
Respiratory rate, breaths per minute, median (IQR)	20 (18, 22)	21 (20, 24)	21 (20, 24)	20 (19, 23)
Missing	7	0	5	12
Oxygen saturation, %, median (IQR)	98 (97, 99)	98 (97, 99)	98 (98, 99)	98 (97, 99)
Missing	1	1	0	2
Heart rate, beats per minute, median (IQR)	86 (80, 96)	87 (73, 100)	90 (76, 104)	86 (78, 100)
Missing	1	1	1	3
Systolic blood pressure, mm Hg, median (IQR)	123 (112, 135)	124 (113, 131)	122 (111, 136)	123 (112, 135)
Missing	7	3	4	14
Glasgow Coma Scale, median (IQR)	15 (15, 15)	15 (15, 15)	15 (15, 15)	15 (15, 15)
Missing	2	1	0	3
Injury Severity Score, median (IQR)	1 (1, 8)	4 (1, 5)	4 (1, 8)	4 (1, 8)
Missing	37	5	35	77
In-hospital mortality, n (%)	21 (11%)	1 (2%)	7 (5%)	29 (8%)
Missing	2	1	0	3
30-day mortality, n (%)	23 (13%)	1 (3%)	8 (7%)	32 (10%)
Missing	29	4	20	53

Missing data counts are only shown for variables with missing values. The absence of a count indicates complete data.

ATLS, Advanced Trauma Life Support; PTC, Primary Trauma Care; SBP, systolic blood pressure.

After training, a total of 22 (16%) patients in the standard care arm died within 30 days, compared with 1 (4%) patient in the ATLS arm and 3 (5%) patients in the PTC arm. The corresponding rates for in-hospital mortality were 19 (12%), 1 (4%) and 3 (4%) for the standard care, ATLS and PTC arms, respectively.

### Outcomes

The percentage of patients who consented to follow-up was 77% and the percentage lost to follow-up was 14%. The missing data rate ranged from 0% to 50%, with details for selected variables shown in table 1 and in online supplemental Table S2. The variables with the largest amount of missing data were the cost of treatment, complications and cause of death, also reported in online supplemental Table S2.

Differences in distributions between directly observed data and data extracted from medical records for selected variables collected through observation or interviews are shown in table 2. Overall, the data were similarly distributed, but there were considerably more missing values in the data extracted from medical records than in the directly observed data.

**Table 2:** Differences in distributions between directly observed data and data extracted from medical records, for selected variables collected through observation or interview in a convenience sample of patients

Characteristic	Directly observed N=55	Medical records N=55
Age, years, median (IQR)	34 (27, 48)	34 (25, 50)
Missing	0	22
Sex, n (%)		
Female	10 (18%)	6 (18%)
Male	45 (82%)	27 (82%)
Missing	0	22
Dominating injury type, n (%)		
Blunt	52 (95%)	29 (91%)
Penetrating	3 (5%)	3 (9%)
Missing	0	23
Respiratory rate, breaths per minute, median (IQR)	21 (18, 24)	18 (16, 20)
Missing	0	37
Oxygen saturation, %, median (IQR)	98 (98, 99)	98 (97, 100)
Missing	0	29
Heart rate, beats per minute, median (IQR)	85 (80, 98)	87 (84, 93)
Missing	0	19
Systolic blood pressure, mm Hg, median (IQR)	123 (112, 136)	118 (110, 128)
Missing	1	18

The percentage of residents who consented to training was 100% and the pass rate was also 100%.

## Discussion

We demonstrated that it is feasible to conduct and collect data for a cluster randomised controlled trial comparing ATLS with PTC and standard care. The missing data rate was low for key variables. However, some variables had very high missing data rates and may not be feasible to include in a full-scale trial, or may require different data collection methods. The distribution of data extracted from medical records was similar to the distribution of directly observed data, but there were more missing values among the extracted data, suggesting that data collected from medical records are reliable even if they are less complete. To increase the completeness of data extracted from the medical records, a full-scale trial should limit the number of variables extracted from medical records and emphasise the importance of having these variables recorded to the participating hospitals.

All-cause 30-day mortality data were missing for 14% of patients. This rate may be high, especially compared with, for example, the CRASH-2 and REACT-2 trials, which reported missing primary outcomes for fewer than 0.01% of patients.^31 32^ Like many other trauma trials, both CRASH-2 and REACT-2 used in-hospital mortality as their primary outcome measure, whereas we attempted to follow patients after discharge. Our missing data rate for in-hospital mortality was only 1%, which is comparable to those in previous trials. Following patients after discharge is notoriously challenging in this setting, and the full-scale trial may need to focus on in-hospital mortality as the primary outcome.

During this pilot study, we deviated from the protocol in several ways. The most significant deviation was a revision of the study aim, as we initially intended to estimate potential effect sizes and other parameters to help sample size calculations for a full-scale trial, in addition to assessing the feasibility outcomes. However, current guidance advises against using pilot studies to estimate effect sizes, as the usefulness of these estimates is questionable.^25 33^ We, therefore, chose to report patient outcomes descriptively. Another significant deviation was the training of emergency medicine residents. We originally planned to train only surgical residents, but trauma management routines varied between participating hospitals, and we adapted to local routines. A full-scale study will need to accommodate this variation as part of the protocol.

There are several significant limitations of this pilot study and, therefore, additional lessons to be learnt and factored into the design of a full-scale trial. First, the patient volumes at some of the participating hospitals were lower than expected. A careful assessment of patient volumes as part of the screening process should be included for a full-scale trial. Second, data on complications and causes of death were almost universally missing. Collecting data on these variables will require alternative methods, as these were not explicitly described in the medical records and autopsy reports were not readily available. Third, we did not collect detailed data on the standard care at each hospital. These data should be collected as part of the screening process for a full-scale trial. Fourth, we used sealed envelopes for randomisation, which increases the risk of bias and errors. A full-scale trial should use a computer-generated randomisation system. Fifth, we did not blind the data analysts, but recommend doing so in a full-scale trial. Sixth, we assessed a large number of potential outcomes, and a full-scale trial should focus on the most relevant outcomes. Seventh, the follow-up period was only 1 month, and changes in some of the outcomes may take longer than that to manifest. The effect on the outcomes is also likely to depend on the adherence to the training, which is not assessed in this pilot study. Finally, owing to a data uploading error, limited data were available from two clusters. At the time of data collection, network and other technical issues were present in some of the clusters, which could explain this error. Regardless, this is a major concern that must be mitigated in a full-scale trial by using a more robust data collection system with local offline backups and careful centralised monitoring.

Previous studies on the effects of ATLS or PTC training on patient outcomes have been observational or quasi-experimental without a control group, with heterogeneous results.^10^,^2234 35^ Most suggest that these programmes are associated with improved outcomes, although not all report significant effects.^10^,^20^ In contrast, some studies have shown potential associations with increased mortality.^21 22^ We observed fewer deaths in the intervention arms than in the standard care arm. This difference may have resulted from the randomisation process with a small number of heterogeneous clusters, highlighting the importance of taking varying cluster sizes into account when designing a full-scale trial.

A full-scale trial remains ethically justifiable after this pilot study, considering that it was never powered to detect meaningful differences in clinical outcomes. In addition, educating physicians in trauma life support through programmes such as ATLS and PTC is considered standard care in many settings, but this approach has been criticised for being costly and for propagating outdated practices.^36^ Several systematic reviews have called for trials in settings where these programmes are not routinely implemented.^2^,^4^ In recognition of their widespread use and high face validity, a stepped-wedge design in which all clusters receive the intervention but at randomised time points may be the best trial design. With regard to generalisability, the study was conducted in India, and the results are likely to be generalisable to other settings with similar trauma care systems. The findings may be less generalisable to settings where senior faculty are more directly involved in the initial management of trauma patients, as these were not trained in this pilot study.

Our study represents the first published attempt to pilot a controlled trial evaluating the effect of ATLS and PTC on patient outcomes. We conclude that a full-scale cluster randomised trial is feasible after incorporating the lessons of this pilot study.

## Supplementary material

10.1136/bmjopen-2025-099020 online supplemental file 1

## Data Availability

The code for the analysis is released publicly on GitHub (https://github.com/martingerdin/tern-pilot). The final anonymised dataset is available from the corresponding author on request.
