# Supplementary material for: Feasibility of a cluster randomised trial on the effect of trauma life support training: a pilot study in India
Source: BMJ Open. 2025 Dec 10;15(12):e099020. doi: 10.1136/bmjopen-2025-099020 (PMC12699583; doi:10.1136/bmjopen-2025-099020)
Supplement: online supplemental file 1 [file bmjopen-15-12-s001.docx]

Supplementary Material

Feasibility of a Cluster Randomised Trial on the Effect of Trauma Life Support Training: A Pilot Study in India

Trauma life support training Effectiveness Research Network (TERN) collaborators

Table of Contents

[S1 Protocol Deviations 1](#_Toc205201238)

[Table S1. Cluster characteristics 2](#_Toc205201239)

[Table S2. Extended patient sample characteristics 3](#_Toc205201240)

# S1 Protocol Deviations

## Trial Registration

We intended to register our trial with Clinical Trials Registry - India (CTRI), but because of a communication error in the management group this was only attempted after the trial has started including patients, and CTRI only accept prospective registrations. We intend to register the full scale trial with both ClinicalTrials.gov and CTRI.

## Aim

In the original protocol, we also aimed to estimate probable effect sizes and other measures needed for the sample size calculations of a full-scale trial, but we revised this aim in the light of current guidance on the conduct and reporting of pilot trials

## Number of Participating Clusters

We recruited seven hospitals as clusters instead of six, as a seventh hospital expressed interest and we had the budget to accommodate this request. We therefore assigned two centres each to the intervention arms and three centres to the control arm.

## Resident Participants

We included emergency medicine residents in addition to surgical residents.

## Periodic surveys of residents

We did not distribute periodic surveys to the participating residents but discussed challenges and suggestions that they had regarding the scheduling or implementation of the training programs.

## Follow up of residents

We stated that resident participants would be followed up 30 days after training, but revised this to follow them up after the end of the study period.

## Data collection from records

We decided to extract data from medical records only for a convenience sample of patients to reduce the research officers’ workload.

## Selection of units for training

We planned to use simple random sampling to select units if there were more than two eligible units in a hospital but instead the hospital principal investigator decided which units to train.

## Timing of resident consent

We had initially planned to ask residents for consent before randomisation, but because of logistical issues the units were only finalised after the hospitals had been randomised. Residents were therefore approached for consent after randomisation but before training.

## Analysis level of feasibility outcomes

We had planned to analyse feasibility outcomes on both an overall and individual cluster level, but we only analysed them on an overall level, because the sample sizes in individual clusters were too small to generate meaningful results.

## Patient outcomes across subgroups

Because of small numbers in the pre-specified subgroups men, women, blunt multisystem trauma, penetrating trauma, shock, severe traumatic brain injury and elderly we decided to report only descriptive data on these subgroups.

# Table S1. Cluster characteristics

| Cluster | City | Beds | Type |
| --- | --- | --- | --- |
| Dr R N Cooper Municipal General Hospital | Mumbai | 600 | Public |
| Seth GS Medical College and KEM Hospital | Mumbai | 1800 | Public |
| Lokmanya Tilak Municipal Medical College and General Hospital | Mumbai | 1850 | Public |
| MEDICA Superspeciality Hospital | Kolkata | 300 | Private |
| Medical College | Kolkata | 2140 | Public |
| Nilratan Sircar Medical College & Hospital | Kolkata | 1910 | Public |
| Postgraduate Institute of Medical Education and Research | Chandigarh | 1400 | Public |

# Table S2. Extended patient sample characteristics

|  | Before training | | | | After training | | | | Overall |
| --- | --- | --- | --- | --- | --- | --- | --- | --- | --- |
| **Characteristic** | **Standard care** N = 41*^1^* | **ATLS** N = 16*^1^* | **PTC** N = 57*^1^* | **Overall** N = 114*^1^* | **Standard care** N = 161*^1^* | **ATLS** N = 28*^1^* | **PTC** N = 73*^1^* | **Overall** N = 262*^1^* | **N = 376***^1^* |
| Age, years | 32 (23, 46) | 46 (30, 61) | 30 (22, 38) | 33 (23, 46) | 35 (26, 47) | 37 (30, 55) | 30 (22, 38) | 34 (25, 45) | 33 (24, 46) |
| Elderly (Age ≥ 65 years) | 3 (7%) | 3 (19%) | 3 (5%) | 9 (8%) | 12 (7%) | 3 (11%) | 2 (3%) | 17 (6%) | 26 (7%) |
| Sex |  |  |  |  |  |  |  |  |  |
| Male | 36 (88%) | 10 (63%) | 44 (77%) | 90 (79%) | 124 (77%) | 23 (82%) | 53 (73%) | 200 (76%) | 290 (77%) |
| Female | 5 (12%) | 6 (38%) | 13 (23%) | 24 (21%) | 37 (23%) | 5 (18%) | 20 (27%) | 62 (24%) | 86 (23%) |
| Dominating injury type |  |  |  |  |  |  |  |  |  |
| Penetrating | 3 (7%) | 2 (13%) | 0 (0%) | 5 (4%) | 10 (6%) | 1 (4%) | 1 (1%) | 12 (5%) | 17 (5%) |
| Blunt | 38 (93%) | 14 (88%) | 57 (100%) | 109 (96%) | 151 (94%) | 27 (96%) | 72 (99%) | 250 (95%) | 359 (95%) |
| Blunt multisystem trauma | 0 (0%) | 1 (6%) | 3 (5%) | 4 (4%) | 2 (1%) | 1 (4%) | 3 (4%) | 6 (2%) | 10 (3%) |
| Severe traumatic brain injury | 3 (8%) | 0 (0%) | 3 (5%) | 6 (5%) | 7 (4%) | 1 (4%) | 2 (3%) | 10 (4%) | 16 (4%) |
| Missing | 1 | 0 | 0 | 1 |  |  |  |  | 1 |
| Shock (SBP ≤ 90 mmHg) | 0 (0%) | 1 (7%) | 0 (0%) | 1 (1%) | 4 (3%) | 1 (4%) | 4 (6%) | 9 (4%) | 10 (3%) |
| Missing | 3 | 1 | 1 | 5 | 4 | 2 | 3 | 9 | 14 |
| Respiratory rate, breaths per minute | 20 (18, 21) | 22 (20, 24) | 21 (19, 23) | 20 (18, 22) | 20 (18, 22) | 21 (19, 24) | 22 (20, 25) | 21 (19, 23) | 20 (19, 23) |
| Missing | 4 | 0 | 3 | 7 | 3 | 0 | 2 | 5 | 12 |
| Oxygen saturation, % | 98 (98, 99) | 98 (96, 99) | 98 (97, 98) | 98 (97, 99) | 98 (97, 99) | 98 (97, 99) | 98 (98, 99) | 98 (98, 99) | 98 (97, 99) |
| Missing | 1 | 1 | 0 | 2 |  |  |  |  | 2 |
| Heart rate, beats per minute | 86 (80, 97) | 94 (74, 106) | 90 (79, 104) | 88 (80, 100) | 85 (80, 95) | 86 (73, 97) | 90 (74, 105) | 86 (78, 100) | 86 (78, 100) |
| Missing | 1 | 0 | 1 | 2 | 0 | 1 | 0 | 1 | 3 |
| Systolic blood pressure, mmHg | 126 (116, 130) | 128 (113, 150) | 123 (115, 138) | 124 (115, 133) | 123 (112, 136) | 124 (113, 130) | 120 (110, 136) | 123 (111, 136) | 123 (112, 135) |
| Missing | 3 | 1 | 1 | 5 | 4 | 2 | 3 | 9 | 14 |
| Glasgow Coma Scale | 15 (15, 15) | 15 (15, 15) | 15 (15, 15) | 15 (15, 15) | 15 (15, 15) | 15 (15, 15) | 15 (15, 15) | 15 (15, 15) | 15 (15, 15) |
| Missing | 1 | 1 | 0 | 2 | 1 | 0 | 0 | 1 | 3 |
| Injury Severity Score | 1 (1, 4) | 4 (1, 25) | 4 (2, 16) | 4 (1, 14) | 4 (1, 9) | 4 (1, 4) | 4 (1, 5) | 4 (1, 6) | 4 (1, 8) |
| Missing | 5 | 2 | 18 | 25 | 32 | 3 | 17 | 52 | 77 |
| In-hospital mortality | 2 (5%) | 0 (0%) | 4 (7%) | 6 (5%) | 19 (12%) | 1 (4%) | 3 (4%) | 23 (9%) | 29 (8%) |
| 30 day mortality | 1 (3%) | 0 (0%) | 5 (10%) | 6 (6%) | 22 (16%) | 1 (4%) | 3 (5%) | 26 (12%) | 32 (10%) |
| Missing | 3 | 2 | 8 | 13 | 26 | 2 | 12 | 40 | 53 |
| 24 hour mortality | 1 (2%) | 0 (0%) | 0 (0%) | 1 (1%) | 0 (0%) | 0 (0%) | 0 (0%) | 0 (0%) | 1 (0%) |
| Missing | 0 | 2 | 5 | 7 | 7 | 2 | 8 | 17 | 24 |
| Self-ambulatory at discharge | 37 (95%) | 11 (92%) | 47 (96%) | 95 (95%) | 135 (99%) | 24 (100%) | 68 (97%) | 227 (98%) | 322 (97%) |
| Missing | 2 | 4 | 8 | 14 | 24 | 4 | 3 | 31 | 45 |
| Return to work | 12 (41%) | 5 (83%) | 31 (74%) | 48 (62%) | 56 (53%) | 11 (58%) | 40 (70%) | 107 (59%) | 155 (60%) |
| Missing | 12 | 10 | 15 | 37 | 56 | 9 | 16 | 81 | 118 |
| Pulmonary complication | 0 (0%) | 0 (0%) | 1 (2%) | 1 (1%) | 0 (0%) | 0 (0%) | 0 (0%) | 0 (0%) | 1 (0%) |
| Missing | 2 | 6 | 11 | 19 | 33 | 5 | 10 | 48 | 67 |
| Septic complication | 1 (3%) | 0 (0%) | 2 (4%) | 3 (3%) | 1 (1%) | 0 (0%) | 2 (3%) | 3 (1%) | 6 (2%) |
| Missing | 1 | 6 | 12 | 19 | 33 | 5 | 10 | 48 | 67 |
| Renal failure | 0 (0%) | 0 (0%) | 0 (0%) | 0 (0%) | 0 (0%) | 0 (0%) | 0 (0%) | 0 (0%) | 0 (0%) |
| Missing | 1 | 5 | 11 | 17 | 32 | 5 | 10 | 47 | 64 |
| Coagulopathy | 0 (0%) | 0 (0%) | 0 (0%) | 0 (0%) | 0 (0%) | 1 (4%) | 0 (0%) | 1 (0%) | 1 (0%) |
| Missing | 1 | 6 | 12 | 19 | 32 | 5 | 10 | 47 | 66 |
| Need for reexploration or resurgery | 0 (0%) | 0 (0%) | 1 (2%) | 1 (1%) | 3 (3%) | 0 (0%) | 1 (2%) | 4 (2%) | 5 (2%) |
| Missing | 1 | 4 | 10 | 15 | 42 | 6 | 10 | 58 | 73 |
| Failure of conservative management | 0 (0%) | 0 (0%) | 1 (2%) | 1 (1%) | 2 (2%) | 2 (8%) | 1 (2%) | 5 (2%) | 6 (2%) |
| Missing | 2 | 10 | 11 | 23 | 44 | 3 | 12 | 59 | 82 |
| EQ-5D mobility at discharge |  |  |  |  |  |  |  |  |  |
| I have no problems in walking | 24 (62%) | 8 (67%) | 34 (83%) | 66 (72%) | 73 (62%) | 16 (89%) | 55 (92%) | 144 (73%) | 210 (73%) |
| I have some problems in walking | 7 (18%) | 1 (8%) | 5 (12%) | 13 (14%) | 22 (19%) | 2 (11%) | 3 (5%) | 27 (14%) | 40 (14%) |
| I am confined to bed | 8 (21%) | 3 (25%) | 2 (5%) | 13 (14%) | 23 (19%) | 0 (0%) | 2 (3%) | 25 (13%) | 38 (13%) |
| Missing | 2 | 4 | 16 | 22 | 43 | 10 | 13 | 66 | 88 |
| EQ-5D self-care at discharge |  |  |  |  |  |  |  |  |  |
| I have no problems with self-care | 23 (59%) | 7 (58%) | 33 (80%) | 63 (68%) | 59 (50%) | 12 (67%) | 51 (85%) | 122 (62%) | 185 (64%) |
| I have some problems bathing or dressing myself | 6 (15%) | 2 (17%) | 5 (12%) | 13 (14%) | 36 (31%) | 2 (11%) | 5 (8%) | 43 (22%) | 56 (19%) |
| I am unable to bathe or dress myself | 10 (26%) | 3 (25%) | 3 (7%) | 16 (17%) | 23 (19%) | 4 (22%) | 4 (7%) | 31 (16%) | 47 (16%) |
| Missing | 2 | 4 | 16 | 22 | 43 | 10 | 13 | 66 | 88 |
| EQ-5D usual activities at discharge |  |  |  |  |  |  |  |  |  |
| I have no problems in performing my usual activities | 21 (54%) | 6 (50%) | 31 (76%) | 58 (63%) | 55 (47%) | 11 (61%) | 51 (85%) | 117 (60%) | 175 (61%) |
| I have some problems in performing my usual activities | 11 (28%) | 3 (25%) | 6 (15%) | 20 (22%) | 45 (38%) | 4 (22%) | 5 (8%) | 54 (28%) | 74 (26%) |
| I am unable to perform my usual activities | 7 (18%) | 3 (25%) | 4 (10%) | 14 (15%) | 18 (15%) | 3 (17%) | 4 (7%) | 25 (13%) | 39 (14%) |
| Missing | 2 | 4 | 16 | 22 | 43 | 10 | 13 | 66 | 88 |
| EQ-5D pain/discomfort at discharge |  |  |  |  |  |  |  |  |  |
| I have no pain or discomfort | 13 (33%) | 4 (33%) | 14 (34%) | 31 (34%) | 30 (25%) | 9 (50%) | 39 (65%) | 78 (40%) | 109 (38%) |
| I have moderate pain or discomfort | 16 (41%) | 8 (67%) | 26 (63%) | 50 (54%) | 41 (35%) | 9 (50%) | 18 (30%) | 68 (35%) | 118 (41%) |
| I have extreme pain or discomfort | 10 (26%) | 0 (0%) | 1 (2%) | 11 (12%) | 47 (40%) | 0 (0%) | 3 (5%) | 50 (26%) | 61 (21%) |
| Missing | 2 | 4 | 16 | 22 | 43 | 10 | 13 | 66 | 88 |
| EQ-5D anxiety/depression at discharge |  |  |  |  |  |  |  |  |  |
| I am not anxious or depressed | 31 (79%) | 6 (50%) | 30 (73%) | 67 (73%) | 108 (92%) | 15 (83%) | 57 (95%) | 180 (92%) | 247 (86%) |
| I am moderately anxious or depressed | 7 (18%) | 6 (50%) | 10 (24%) | 23 (25%) | 8 (7%) | 3 (17%) | 3 (5%) | 14 (7%) | 37 (13%) |
| I am extremely anxious or depressed | 1 (3%) | 0 (0%) | 1 (2%) | 2 (2%) | 2 (2%) | 0 (0%) | 0 (0%) | 2 (1%) | 4 (1%) |
| Missing | 2 | 4 | 16 | 22 | 43 | 10 | 13 | 66 | 88 |
| EQ-5D mobility at 30 day follow-up |  |  |  |  |  |  |  |  |  |
| I have no problems in walking | 20 (65%) | 4 (67%) | 39 (91%) | 63 (79%) | 79 (77%) | 16 (80%) | 57 (98%) | 152 (84%) | 215 (83%) |
| I have some problems in walking | 4 (13%) | 1 (17%) | 3 (7%) | 8 (10%) | 17 (17%) | 4 (20%) | 0 (0%) | 21 (12%) | 29 (11%) |
| I am confined to bed | 7 (23%) | 1 (17%) | 1 (2%) | 9 (11%) | 6 (6%) | 0 (0%) | 1 (2%) | 7 (4%) | 16 (6%) |
| Missing | 10 | 10 | 14 | 34 | 59 | 8 | 15 | 82 | 116 |
| EQ-5D self-care at 30 day follow-up |  |  |  |  |  |  |  |  |  |
| I have no problems with self-care | 22 (71%) | 5 (83%) | 39 (91%) | 66 (83%) | 81 (79%) | 16 (80%) | 56 (97%) | 153 (85%) | 219 (84%) |
| I have some problems bathing or dressing myself | 2 (6%) | 1 (17%) | 2 (5%) | 5 (6%) | 15 (15%) | 2 (10%) | 0 (0%) | 17 (9%) | 22 (8%) |
| I am unable to bathe or dress myself | 7 (23%) | 0 (0%) | 2 (5%) | 9 (11%) | 6 (6%) | 2 (10%) | 2 (3%) | 10 (6%) | 19 (7%) |
| Missing | 10 | 10 | 14 | 34 | 59 | 8 | 15 | 82 | 116 |
| EQ-5D usual activities at 30 day follow-up |  |  |  |  |  |  |  |  |  |
| I have no problems in performing my usual activities | 22 (71%) | 6 (100%) | 39 (91%) | 67 (84%) | 83 (81%) | 15 (75%) | 55 (95%) | 153 (85%) | 220 (85%) |
| I have some problems in performing my usual activities | 6 (19%) | 0 (0%) | 2 (5%) | 8 (10%) | 12 (12%) | 3 (15%) | 1 (2%) | 16 (9%) | 24 (9%) |
| I am unable to perform my usual activities | 3 (10%) | 0 (0%) | 2 (5%) | 5 (6%) | 7 (7%) | 2 (10%) | 2 (3%) | 11 (6%) | 16 (6%) |
| Missing | 10 | 10 | 14 | 34 | 59 | 8 | 15 | 82 | 116 |
| EQ-5D pain/discomfort at 30 day follow-up |  |  |  |  |  |  |  |  |  |
| I have no pain or discomfort | 23 (74%) | 4 (67%) | 33 (77%) | 60 (75%) | 81 (79%) | 16 (80%) | 50 (86%) | 147 (82%) | 207 (80%) |
| I have moderate pain or discomfort | 8 (26%) | 2 (33%) | 9 (21%) | 19 (24%) | 19 (19%) | 4 (20%) | 8 (14%) | 31 (17%) | 50 (19%) |
| I have extreme pain or discomfort | 0 (0%) | 0 (0%) | 1 (2%) | 1 (1%) | 2 (2%) | 0 (0%) | 0 (0%) | 2 (1%) | 3 (1%) |
| Missing | 10 | 10 | 14 | 34 | 59 | 8 | 15 | 82 | 116 |
| EQ-5D anxiety/depression at 30 day follow-up |  |  |  |  |  |  |  |  |  |
| I am not anxious or depressed | 30 (97%) | 6 (100%) | 37 (86%) | 73 (91%) | 97 (95%) | 17 (85%) | 56 (97%) | 170 (94%) | 243 (93%) |
| I am moderately anxious or depressed | 1 (3%) | 0 (0%) | 5 (12%) | 6 (8%) | 4 (4%) | 3 (15%) | 2 (3%) | 9 (5%) | 15 (6%) |
| I am extremely anxious or depressed | 0 (0%) | 0 (0%) | 1 (2%) | 1 (1%) | 1 (1%) | 0 (0%) | 0 (0%) | 1 (1%) | 2 (1%) |
| Missing | 10 | 10 | 14 | 34 | 59 | 8 | 15 | 82 | 116 |
| Patient satisfaction |  |  |  |  |  |  |  |  |  |
| Very satisfied | 26 (67%) | 9 (64%) | 37 (73%) | 72 (69%) | 97 (70%) | 19 (79%) | 66 (94%) | 182 (78%) | 254 (76%) |
| Somewhat satisfied | 9 (23%) | 5 (36%) | 8 (16%) | 22 (21%) | 20 (14%) | 3 (13%) | 4 (6%) | 27 (12%) | 49 (15%) |
| Somewhat dissatisfied | 3 (8%) | 0 (0%) | 2 (4%) | 5 (5%) | 13 (9%) | 1 (4%) | 0 (0%) | 14 (6%) | 19 (6%) |
| Very dissatisfied | 1 (3%) | 0 (0%) | 4 (8%) | 5 (5%) | 8 (6%) | 1 (4%) | 0 (0%) | 9 (4%) | 14 (4%) |
| Missing | 2 | 2 | 6 | 10 | 23 | 4 | 3 | 30 | 40 |
| Number of hospitalizations for this injury | 0 (0%) | 1 (17%) | 3 (6%) | 4 (4%) | 6 (5%) | 0 (0%) | 0 (0%) | 6 (3%) | 10 (3%) |
| Missing | 2 | 10 | 8 | 20 | 34 | 3 | 11 | 48 | 68 |
| EQ-5D health state at discharge | 50 (3, 90) | 60 (40, 90) | 80 (60, 90) | 75 (40, 90) | 50 (4, 80) | 65 (50, 80) | 90 (79, 100) | 70 (5, 90) | 70 (10, 90) |
| Missing | 10 | 5 | 16 | 31 | 48 | 10 | 19 | 77 | 108 |
| EQ-5D health state at 30 day follow-up | 10 (7, 100) | 100 (60, 100) | 100 (89, 100) | 99 (70, 100) | 83 (9, 100) | 83 (70, 100) | 100 (95, 100) | 100 (45, 100) | 100 (50, 100) |
| Missing | 14 | 10 | 15 | 39 | 61 | 8 | 17 | 86 | 125 |
| Cost of treatment | 14,000 (500, 53,000) | 7,000 (0, 14,000) | 2,025 (500, 11,000) | 3,000 (500, 15,000) | 2,500 (500, 25,000) | 5,000 (0, 15,000) | 1,500 (200, 3,000) | 2,000 (299, 13,000) | 2,000 (500, 14,000) |
| Missing | 23 | 14 | 18 | 55 | 90 | 21 | 21 | 132 | 187 |
| *^1^*Median (Q1, Q3); n (%) | | | | | | | | | |
