# Supplementary material for: Feasibility of a cluster randomised trial on the effect of trauma life support training: a pilot study in India
Source: BMJ Open. 2025 Dec 10;15(12):e099020. doi: 10.1136/bmjopen-2025-099020 (PMC12699583; doi:10.1136/bmjopen-2025-099020)
Supplement: Supplementary file 2 [file bmjopen-2025-099020.reviewer_comments.pdf]

PEER REVIEW HISTORY

BMJ Open publishes all reviews undertaken for accepted manuscripts. Reviewers are asked to complete a checklist review form and are provided with free text boxes to elaborate on their assessment. These free text comments are reproduced below.

ARTICLE DETAILS

Title (Provisional)

Feasibility of a Cluster Randomised Trial on the Effect of Trauma Life Support Training: A Pilot Study in India

Authors

Gerdin Wärnberg, Martin; Basak, Debojit; Berg, Johanna; Chatterjee, Shamita; Felländer-Tsai, Li; Ghag, Geeta; Juillard, Catherine; Khajanchi, Monty; Khan, Tamal; Nandu, Vipul; Roy, Nobhojit; Singh, Rajdeep; Soni, Kapil Dev; Strömmer, Lovisa

---

VERSION 1 - REVIEW

**Reviewer**	**1**
**Name**	**Sidhwa , Feroze**
**Affiliation**	**San Joaquin General Hospital, Surgery and trauma**
**Date**	**21-Feb-2025**
**COI**	**None**

"We found that the ATLS ® and PTC arms had lower 30-day mortality compared to the PTC and standard care arms. This finding could hint towards a potential effect of training physicians in trauma life support, but it is important to note that this pilot study was not powered to detect any differences in outcomes. The arms differed considerably in sample size, with the ATLS ® arm having the smallest sample size. This difference most likely resulted from the randomisation process with a small number of heterogeneous clusters, and this heterogeneity highlights the importance of taking varying cluster sizes into account in the design of the full scale trial."

If this is the case then these outcomes simply should not be reported. Since this is a feasibility trial the major concern should be assessing missing data, which it seems to be, but this statement will be controversial and may distract from the otherwise important and better-supported conclusions. I would suggest removing discussion and reporting of mortality, and instead simply report on whether or not mortality data was missing, and if so was it differentially missing in the different arms of the study. If the study is not powered to detect a difference and no measures of statistical significance were calculated then this is not useful information to the reader.

The above is the only concern I had when I chose "no" for "Are the discussion and conclusions justified by the results?"

**Reviewer**	**2**
**Name**	**Ham, Wietske H.W.**
**Affiliation**	**University of Applied Sciences**
**Date**	**06-Mar-2025**
**COI**	**None**

Thank you for submitting this relevant paper and the impressive work behind it. It was an honor to review the paper. Here are some suggestions and comments, which I hope will be helpful in further developing the paper. The aim of the study is to gain insight into the feasibility of a larger study. At the same time, more results are presented than necessary to answer this question. This is not clear to the reader. It is unclear to me whether patients who ultimately did not regain consciousness were also included in this study. This should be described more clearly. It also seems that, given the research period, the study sites were included sequentially; if so, the order and period should be described. It is not sufficiently clear to me how the statistical methods used contribute to answering the research question, namely the feasibility of an RCT and determining an effect size (and other measures, which are too vague). It seems that in the description of this manuscript, the pilot outcomes are mixed with the outcomes of the trial itself, which is confusing. This is likely due to the way it is written (it needs to be better explained and specified): the authors describe results, but also the outcomes. It is unclear in the current writing how the outcomes contribute to answering the research question. The flow diagram does not clearly show how many participants did not participate or were missed. The design of the study itself seems to have been conducted properly. In the discussion, deviations from the protocol are mentioned and referred to in the appendix, while these data are actually appropriate to discuss (and share) in the article itself, since it is a pilot. I find the number of appendices in the appendix very extensive, which makes it lose its purpose, and I also wonder to what extent it contributes to answering the research question. Parts of the protocol, which are now included in the appendix, should, in my opinion, be incorporated into the main paper.

**Reviewer**	**3**
**Name**	**Sprague, Sheila**
**Affiliation**	**McMaster University**
**Date**	**03-Apr-2025**

COI **None**

Thank you for the opportunity to review the manuscript titled "Feasibility of a Cluster Randomised Trial on the Effect of Trauma Life Support Training: A Pilot Study". This manuscript describes an important trial with the potential to save many lives at hospitals across India. There are many sections in this manuscript which are unclear. The overall structure of the manuscript also needs to be improved upon. Detailed comments are below.

Introduction

- Provide clear objectives for the both the pilot phase and the definitive trial phase

Methods

- Describe what information is being collected on standard of care at each hospital as it varies.

- Provide additional details on ATLS and PTC training interventions.

- Include details on the cluster selection.

- The authors should comment on the sample size for a pilot study. For example, why did they select seven clusters? They should also comment on the number of residents and patients required to allow for a meaningful analysis of the feasibility outcomes.

- The reason for the one-month lead in is unclear. It is unclear how this data will be analyzed.

- Using sealed envelopes for randomization increases the risk of selection bias, tampering, and accidental loss, potentially compromising allocation concealment. Authors should have used the computer-generated randomization system at time of randomization for each site.

- For blinding, the authors should state that it was not possible to blind those involved. It is unclear if those analyzing the data were blinded, as this is possible.

- The follow-up period for each participant is unclear. The methods state that data were collected for four months. The methods also mention a 3-day mortality outcome.

- Success criteria and threshold details for the feasibility outcomes should be clearly outlined in the methods.

- The outcomes for the definitive trial (clinical outcomes) need to be clearly listed and described.

- The statistical analysis section is unclear. It should list the descriptive analyses plans and then the analyses used to assess the feasibility objectives. Feasibility pilot studies are not powered to detect meaningful differences between groups. Rather their goal is to determine whether a definitive trial is practical by assessing feasibility metrics. The clinical outcomes (the outcomes for the definitive trial) could be reported descriptively, but the use of comparative statistics (differences in final values, differences from baseline, confidence intervals, and subgroup analyses) to assess these outcomes is not appropriate during the

pilot phase and potentially misleading, especially without a formal sample size calculation being conducted.

- The statistical analyses section also describes inconsistent times for outcome comparisons. The intervention arms measure change from baseline over one month pre-training vs. three months post-training, while the control arm measures change over a four-month period. For the definitive trial, the timing of assessment should be consistent across all arms to avoid biased comparisons.

Results

- Add a description of each cluster.

- Comment on why the number of residents and patients varied across the treatment groups.

- A few key prognostic factors differ across the treatment groups (age, ISS).

- Consider reporting overall enrollment rates (e.g. number of participants enrolled per month). There is a paragraph in the discussion on low enrollment, but it is not listed in the results.

- The results of feasibility outcomes should be reported clearly in a table, with a column indicating whether the feasibility outcome was successfully achieved.

- Mortality should only be reported once in the results section.

- Report the resident outcomes and the participant outcomes separately.

- List the protocol deviations in the results section, not in the discussion.

- It is unclear the importance and relevance of the before training after training data (Table S1).

- The EQ-5D should be scored as opposed to showing the results for each question.

- Simplify the outcomes tables to focus on the key findings. Remove the yes/no and only list the yes.

- The absolute difference versus relative difference does not add to the analyses and could be removed.

- As this a pilot study, outcome data should be reported descriptively in a clearer manner.

- There are too many outcomes.

Discussion

- Remove all results from the discussion and place them in the results section, then discuss the results in the discussion section.

- Discuss the implication of adding the seventh site in more detail.

- Discuss if there are methods to improve participant follow-up for the definitive trial.

- Discuss if there are methods to reduce missing data for the definitive phase.

- Comment on the ethics of the full trial. Is it ethical to have sites continue with usual care after seeing the pilot study results?

- Consider a stepped wedge design where all clusters start with standard of care and then transition into an intervention.

- Use the CONSORT statement extension for pilot and feasibility trials (https://www.equator-network.org/reporting-guidelines/consort-2010-statement-extension-to-randomised-pilot-and-feasibility-trials/) and the CONSORT statement extension for cluster randomised trials (https://www.equator-network.org/reporting-guidelines/consort-cluster/).

**Reviewer**	**4**
**Name**	**Dack, Kyle**
**Affiliation**	**University of Bristol, MRC Integrative Epidemiology Unit**
**Date**	**09-Jun-2025**
**COI**	**None**

Statistical review summary:

This is a well written paper, with transparent and thorough methodological reporting, and a reliable statistical approach. The purpose of the analysis was to inform a decision on the feasability of a full trial, and as such the results are interpreted with appropriate caution.

I have only minor comments about certain study design aspects which I found unclear - particularly the observed/extracted data comparison.

Methods:

1. Intervention – how long was the training programme, and who was it delivered by? Was the training performed in stages, e.g. to different cohorts of residents within the hospital, or all at once? The first paragraph stated that there is a 1 month observation phase followed by a 3 month intervention phase, but its not clear to me if this means the intervention continued over 3 months, or if the intervention was applied at the beginning of this phase, followed by ~3 months of patient follow-up. Further intervention in the study design and intervention sections could clarify this.

2. Variables – more information on the injury severity score would be useful to readers, because I am unsure how this variable was planned to be used (it is not an outcome), and how precisely it was calculated. A reference is provided, but a high-level overview of the

approach (including the range of potential scores) would help readers understand this measurement better.

3. “Differences in distributions of observed and extracted data” – I am unsure how observed data differs from extracted data – these terms were not previously explained or defined. Does it mean the observation period vs the 3 month intervention period? “distributions” is also somewhat unclear, histogram distributions, or a specific tendency statistic?

4. Statistical analysis: “We used an empty generalised linear mixed model to estimate the intracluster correlation coefficient” which outcomes does this apply to?

5. The analysis involved comparing the 1st month and 2nd-4th months, but it is not described how outcomes will be summed – e.g. consent rate, I imagine this the total consent rate in month 1 vs months 2-4? It would be helpful to directly confirm this in the text.

Results

6. Table 1. Missing counts are reported for some but not all measurements. Does the absence of a count indicate complete data? If so, a footnote stating this would help readers.

7. Table 2. As mentioned in a prior comment it is unclear to me how these two groups are defined. Also, both have an N of 55, assuming these are patients it means there is a total N of 110, less than the total of 376. Why do the numbers differ?

8. Table S1. Suggest clarifying that this is patient characteristics in the title.

9. Table S5. Certain cells are blank – a footnote should explain what this means (no observations?)

VERSION 1 - AUTHOR RESPONSE

Response to Reviewer 1, Dr. Feroze Sidhwa, San Joaquin General Hospital:
Q1.1 “We found that the ATLS ® and PTC arms had lower 30-day mortality compared to the PTC and standard care arms. This finding could hint towards a potential effect of training physicians in trauma life support, but it is important to note that this pilot study was not powered to detect any differences in outcomes. The arms differed considerably in sample size, with the ATLS ® arm having the smallest sample size. This difference most likely resulted from the randomisation process with a small number of heterogeneous clusters, and this heterogeneity highlights the importance of taking varying cluster sizes into account in the design of the full scale trial.”
If this is the case then these outcomes simply should not be reported. Since this is a feasibility trial the major concern should be assessing missing data, which it seems to be, but this statement will be controversial and may distract from the otherwise important and better-supported conclusions. I would suggest removing discussion and reporting of mortality, and instead simply report on whether or not mortality data was missing, and if so was it differentially missing in the different arms of the study. If the study is not powered to detect a difference and no measures of statistical significance were calculated then this is not useful information to the reader.
The above is the only concern I had when I chose "no" for "Are the discussion and conclusions justified by the results?”

R1.1 Thank you for pointing this out and we agree that the reporting of the mortality outcomes is problematic for a feasibility trial. We have decided to report mortality outcomes descriptively in the paper, given comments from other reviewers, but revised the discussion to focus on the missing data and to avoid any conclusions about a potential effect of the training.

Response to Reviewer 2, Dr. Wietske H.W. Ham, University of Applied Sciences:
Q2.1 Thank you for submitting this relevant paper and the impressive work behind it. It was an honor to review the paper. Here are some suggestions and comments, which I hope will be helpful in further developing the paper.

R2.1 Thank you for your comments, they were very helpful. We have revised the manuscript in the light of your comments.

Q2.2 The aim of the study is to gain insight into the feasibility of a larger study. At the same time, more results are presented than necessary to answer this question. This is not clear to the reader.

R2.2 We agree that the manuscript, and the supplementary material, includes more results than necessary to fulfil the aim of the study. As you have noticed, we struggled to find a balance between adhering to the protocol and reporting the results that were most relevant in a transparent manner. We have now attempted to address this by clearly stating that we deviated from the original protocol in reporting the pilot, to focus on the feasibility of the full-scale trial. We have therefore limited the number of results that we present in the main text of the manuscript considerably.

Q2.3 It is unclear to me whether patients who ultimately did not regain consciousness were also included in this study. This should be described more clearly.

R2.3 We have clarified that patients who did not regain consciousness were included based on the consent by their representative.

Q2.4 It also seems that, given the research period, the study sites were included sequentially; if so, the order and period should be described.

R2.4 That is correct, data collection started in each cluster as they received the necessary approvals. The information about the study period in each cluster has been added as supplementary material.

Q2.5 It is not sufficiently clear to me how the statistical methods used contribute to answering the research question, namely the feasibility of an RCT and determining an effect size (and other measures, which are too vague).

R2.5 We have clarified how we used descriptive statistics to summarize patient characteristics and feasibility outcomes. We now describe the methods used for the additional analyses according to the original protocol in the supplementary materials.

Q2.6 It seems that in the description of this manuscript, the pilot outcomes are mixed with the outcomes of the trial itself, which is confusing. This is likely due to the way it is written (it needs to be better explained and specified): the authors describe results, but also the outcomes. It is unclear in the current writing how the outcomes contribute to answering the research question.

R2.6 We agree that this was confusing and have therefore attempted to streamline the paper further by presenting mortality descriptively along with the other patient characteristics, and only report the feasibility outcomes in the outcomes section.

Q2.7 The flow diagram does not clearly show how many participants did not participate or were missed. The design of the study itself seems to have been conducted properly.

R2.7 Thank you for pointing this out. We have added the number of patients who were lost to follow up in each arm to the flow diagram.

Q2.8 In the discussion, deviations from the protocol are mentioned and referred to in the appendix, while these data are actually appropriate to discuss (and share) in the article itself, since it is a pilot.

R2.8 We have revised the manuscript to describe the deviations where they are most relevant, and retained a list in the supplementary materials for completeness.

Q2.9 I find the number of appendices in the appendix very extensive, which makes it lose its purpose, and I also wonder to what extent it contributes to answering the research question. Parts of the protocol, which are now included in the appendix, should, in my opinion, be incorporated into the main paper.

R2.9 We agree that the supplementary materials are very extensive. We included these to transparently report the results of the analyses specified in the original protocol, but in response to your comment we have now published the majority of the supplementary materials as online additional files, and cite these in the manuscript. We have attempted to include parts of the supplementary materials in the main text where appropriate, in response to your earlier comments.

Response to Reviewer 3, Dr. Sheila Sprague, McMaster University:
Q3.1 Thank you for the opportunity to review the manuscript titled "Feasibility of a Cluster Randomised Trial on the Effect of Trauma Life Support Training: A Pilot Study". This manuscript describes an important trial with the potential to save many lives at hospitals across India. There are many sections in this manuscript which are unclear. The overall structure of the manuscript also needs to be improved upon. Detailed comments are below.

R3.1 Thank you for your thorough review. We have made major revisions in response to your comments, and we hope that the manuscript is now clearer.

Introduction
Q3.2 Provide clear objectives for the both the pilot phase and the definitive trial phase

R3.2 We have revised the objective of the pilot study to also include the aim of the full scale trial. The revised objective is "to assess the feasibility of a cluster randomised controlled trial comparing the effect of ATLS® and PTC with standard care on outcomes in adult trauma patients.".

Methods
Q3.3 Describe what information is being collected on standard of care at each hospital as it varies.

R3.3 We did not collect in-depth data on the standard care at each hospital, and we have added a note in the methods section to clarify this. We have also added this as a limitation of this pilot study, and suggested that the full scale trial should include a more detailed assessment of the standard care at each hospital.

Q3.4 Provide additional details on ATLS and PTC training interventions.

R3.4 We have added more details on the ATLS and PTC training interventions to the methods section.

Q3.5 Include details on the cluster selection.

R3.5 We have revised the methods section to clarify that the seven hospitals were a convenience sample of hospitals in India that fulfilled the inclusion criteria, and with existing connections to the research team.

Q3.6 The authors should comment on the sample size for a pilot study. For example, why did they select seven clusters? They should also comment on the number of residents and patients required to allow for a meaningful analysis of the feasibility outcomes.

R3.6 We have added the rationale for the number of clusters and the number of units per cluster to the methods section. We did not conduct a formal power calculation, but deemed that the anticipated sample sizes would be sufficient to evaluate the feasibility outcomes.

Q3.7 The reason for the one-month lead in is unclear. It is unclear how this data will be analysed.

R3.7 We included the one month lead to be able to evaluate the feasibility of comparing patient outcomes both as absolute differences between the intervention phases, and as differences in change from baseline.

Q3.8 Using sealed envelopes for randomization increases the risk of selection bias, tampering, and accidental loss, potentially compromising allocation concealment. Authors should have used the computer-generated randomization system at time of randomization for each site.

R3.8 We recognise that the use of sealed envelopes for randomisation increases the risk of bias and errors, and we have added this as a limitation of this pilot study.

Q3.9 For blinding, the authors should state that it was not possible to blind those involved. It is unclear if those analyzing the data were blinded, as this is possible.

R3.9 We have added that we did not blind the data analysts in this pilot study, but recommend doing so in the full scale trial.

Q3.10 The follow-up period for each participant is unclear. The methods state that data were collected for four months. The methods also mention a 3-day mortality outcome.

R3.10 The data collection period in each hospital was intended to be four months, and then patients were followed up for one month. However, the actual data collected periods varied slightly between clusters, and we have clarified this in the methods section. The 3-day mortality outcome was a typo, and we have corrected this.

Q3.11 Success criteria and threshold details for the feasibility outcomes should be clearly outlined in the methods.

R3.11 We did not specify success criteria for the feasibility outcomes, and we have added this to the methods section.

Q3.12 The outcomes for the definitive trial (clinical outcomes) need to be clearly listed and described.

R3.12 We now specify that we collected data on potential outcomes for the full scale trial, including mortality within 30 days, in-hospital mortality, complications and health related quality of life.

Q3.13 The statistical analysis section is unclear. It should list the descriptive analyses plans and then the analyses used to assess the feasibility objectives. Feasibility pilot studies are not powered to detect meaningful differences between groups. Rather their goal is to determine whether a definitive trial is practical by assessing feasibility metrics. The clinical outcomes (the outcomes for the definitive trial) could be reported descriptively, but the use of comparative statistics (differences in final values, differences from baseline, confidence intervals, and subgroup analyses) to assess these outcomes is not appropriate during the pilot phase and potentially misleading, especially without a formal sample size calculation being conducted.

R3.13 Thank you for pointing this out. We acknowledge that the estimation of potential effect sizes is not appropriate for this pilot study, and we have revised the aim of the study to reflect this and report this change as a deviation from the original protocol. We now report that we used descriptive statistics to analyse the feasibility outcomes and report clinical outcomes descriptively.

Q3.14 The statistical analyses section also describes inconsistent times for outcome comparisons. The intervention arms measure change from baseline over one month pre-training vs. three months post-training, while the control arm measures change over a four-month period. For the definitive trial, the timing of assessment should be consistent across all arms to avoid biased comparisons.

R3.14 We agree.

Results
Q3.15 Add a description of each cluster.

R3.15 We have added a description of each cluster as Supplementary Table S1.

Q3.16 Comment on why the number of residents and patients varied across the treatment groups.

R3.16 We have clarified that the number of residents trained in each intervention cluster depended on the size of the units, and that the number of patients included per cluster varied between clusters depending on the volume of patients at each hospital.

Q3.17 A few key prognostic factors differ across the treatment groups (age, ISS).

R3.17 Thank you for pointing this out and we now highlight this in the results section.

Q3.18 Consider reporting overall enrollment rates (e.g. number of participants enrolled per month). There is a paragraph in the discussion on low enrollment, but it is not listed in the results.

R3.18 We have added a figure to the result's section, reporting the number of patients included per month per cluster. Notably, the process of preparing this figure allowed us to identify a data collection error in two clusters, and we have added this to the methods section. We have also included this in the discussion as a limitation of this pilot study and an important lesson for the full scale trial.

Q3.19 The results of feasibility outcomes should be reported clearly in a table, with a column indicating whether the feasibility outcome was successfully achieved.

R3.19 We have decided not to report the feasibility outcomes in a table, because some of them, for example the comparison of directly observed data and data extracted from medical records, were assessed qualitatively. We have however removed the description of the clinical outcomes from the outcomes section to avoid confusion.

Q3.20 Mortality should only be reported once in the results section.

R3.20 Mortality is now reported together with the other patient characteristics in the results section. We have removed the table comparing clinical outcomes from the results section.

Q3.21 Report the resident outcomes and the participant outcomes separately.

R3.21 We now report the resident outcomes and the participant outcomes separately.

Q3.22 List the protocol deviations in the results section, not in the discussion.

R3.22 We now report the protocol deviations where relevant in the methods section, and include a list as supplementary material for completeness and easy reference.

Q3.23 It is unclear the importance and relevance of the before training after training data (Table S1).

R3.23 We included the one month before training and three month after training data to be able to evaluate the feasibility of comparing patient outcomes both as absolute differences between the intervention phases, and as differences in change from baseline. We have attempted to clarify this in the methods section.

Q3.24 The EQ-5D should be scored as opposed to showing the results for each question.

R3.24 Currently, there is no Indian value set available for the EQ-5D-3L, and we have therefore not scored the data. We now explain this in the methods section.

Q3.25 Simplify the outcomes tables to focus on the key findings. Remove the yes/no and only list the yes.

R3.25 We have removed the majority of the outcome tables from the supplementary materials and published them as online additional files, referenced in the manuscript.

Q3.26 The absolute difference versus relative difference does not add to the analyses and could be removed.

R3.26 We have removed the absolute difference versus relative difference from the main results section, but report them in the additional online files as this analysis was part of the original protocol.

Q3.27 As this a pilot study, outcome data should be reported descriptively in a clearer manner.

R3.27 We have attempted to clarify the reporting of the outcome data in the results section.

Q3.28 There are too many outcomes.

R3.28 We agree and suggest that the full scale trial should focus on the most relevant outcomes.

Discussion
Q3.29 Remove all results from the discussion and place them in the results section, then discuss the results in the discussion section.

R3.29 We now only discuss results already reported in the results section in the discussion.

Q3.30 Discuss the implication of adding the seventh site in more detail.

R3.30 We have clarified that the seventh site was included for pragmatic reasons.

Q3.31 Discuss if there are methods to improve participant follow-up for the definitive trial.

R3.31 We now highlight the challenges of following up patients after discharge in the discussion and suggest that the full scale trial should focus on in-hospital mortality as the primary outcome.

Q3.32 Discuss if there are methods to reduce missing data for the definitive phase.

R3.32 We have added a suggestion to the discussion to improve the completeness of the data extracted from the medical records.

Q3.33 Comment on the ethics of the full trial. Is it ethical to have sites continue with usual care after seeing the pilot study results?

R3.33 We have added a sentence on the ethics of the full trial.

Q3.34 Consider a stepped wedge design where all clusters start with standard of care and then transition into an intervention.

R3.34 Thank you for this suggestion and we agree that a stepped wedge design would be a good approach for the full scale trial. We now include this as a suggestion for the full scale trial in the discussion.

Q3.35 Use the CONSORT statement extension for pilot and feasibility trials (https://www.equator-network.org/reporting-guidelines/consort-2010-statement-extension-to-randomised-pilot-and-feasibility-trials/) and the CONSORT statement extension for cluster randomised trials (https://www.equator-network.org/reporting-guidelines/consort-cluster/).

R3.35 We have attempted to align our reporting with these guidelines.

Response to Reviewer 4, Dr. Kyle Dack, University of Bristol:
This is a well written paper, with transparent and thorough methodological reporting, and a reliable statistical approach. The purpose of the analysis was to inform a decision on the feasability of a full trial, and as such the results are interpreted with appropriate caution.

Q4.1 I have only minor comments about certain study design aspects which I found unclear - particularly the observed/extracted data comparison.

R4.1 Thank you for reviewing the manuscript!

Methods:
Q4.2 Intervention – how long was the training programme, and who was it delivered by? Was the training performed in stages, e.g. to different cohorts of residents within the hospital, or all at once? The first paragraph stated that there is a 1 month observation phase followed by a 3 month intervention phase, but its not clear to me if this means the intervention continued over 3 months, or if the intervention was applied at the beginning of this phase, followed by ~3 months of patient follow-up. Further intervention in the study design and intervention sections could clarify this.

R4.2 We have clarified that the training was conducted at the beginning of the three month intervention phase.

Q4.3 Variables – more information on the injury severity score would be useful to readers, because I am unsure how this variable was planned to be used (it is not an outcome), and how precisely it was calculated. A reference is provided, but a high-level overview of the approach (including the range of potential scores) would help readers understand this measurement better.

R4.3 We have added a description of the ISS to the methods section.

Q4.4 “Differences in distributions of observed and extracted data” – I am unsure how observed data differs from extracted data – these terms were not previously explained or defined. Does it mean the observation period vs the 3 month intervention period? “distributions” is also somewhat unclear, histogram distributions, or a specific tendency statistic?

R4.4 We have attempted to clarify what we mean by distributions (summary statistics) and directly observed data, in the section where we describe the feasibility outcomes.

Q4.5 Statistical analysis: “We used an empty generalised linear mixed model to estimate the intracluster correlation coefficient” which outcomes does this apply to?

R4.5 We have moved the description of this analysis to the supplementary materials, and clarified that it applies to all cause 30-day and in-hospital mortality.

Q4.6 The analysis involved comparing the 1st month and 2nd-4th months, but it is not described how outcomes will be summed – e.g. consent rate, I imagine this the total consent rate in month 1 vs months 2-4? It would be helpful to directly confirm this in the text.

R4.6 We have attempted to clarify that feasibility outcomes were not assessed as differences between the observation and intervention phases, but summarised across the entire period.

Results
Q4.7 Table 1. Missing counts are reported for some but not all measurements. Does the absence of a count indicate complete data? If so, a footnote stating this would help readers.

R4.7 We have added a footnote to Table 1 to clarify that the absence of a count indicates complete data.

Q4.8 Table 2. As mentioned in a prior comment it is unclear to me how these two groups are defined. Also, both have an N of 55, assuming these are patients it means there is a total N of 110, less than the total of 376. Why do the numbers differ?

R4.8 We only extracted data from a convenience sample of patients to reduce the workload of the project officers collecting data. We have attempted to clarify this in the methods section and in the table caption.

Q4.9 Table S1. Suggest clarifying that this is patient characteristics in the title.

R4.9 We have added “patient” to the title.

Q4.10 Table S5. Certain cells are blank – a footnote should explain what this means (no observations?)

R4.10 Yes, that is correct. Empty cells indicate that there were no observations. We have now moved the majority of the tables from the supplementary materials and instead published them as online additional files, referenced in the manuscript. These files include a note to clarify that empty cells indicate that there were no observations.

VERSION 2 - REVIEW

**Reviewer**	**1**
**Name**	**Sidhwa , Feroze**
**Affiliation**	**San Joaquin General Hospital, Surgery and trauma**
**Date**	**09-Aug-2025**
**COI**	

• Improvements in patient outcomes could take much longer than a few months to manifest, and may also be dependent on a certain level of training being achieved in the unit. These limitations should be addressed.

• “The principal investigator at each hospital selected the units for training.” How? Was this based on who conducts the trauma resuscitation?

• How do you know the accuracy of ICD-10 coding at these hospitals?

• Faculty not being included in the study because they are not typically directly involved in the initial management of trauma patients will be a major factor limiting generalizability of the findings. This cannot be helped and not every trial can be generalizable to the entire universe, but this should be noted in future conclusions and limitations.

• “We initially planned to use simple random sampling to select the units to be trained, but for pragmatic reasons this decision was left to the site principal investigator.” Does this mean that the site principal investigator decided what kind of sampling to use, or that they decided on what units to be trained instead of using any kind of randomization?

• An “error in the data uploading process” that led to 1/10th of the data being collected in two out of seven clusters is very alarming. Isn’t it possible this data uploading process error wasn’t a source of random error? For example maybe it drastically reduced the data available from the two most incompetent clusters, regardless of training status? Unless this issue is addressed it would be a major source of potential error in a larger trial. This was mentioned briefly in the conclusion but I would encourage the authors to make clear that they understand that this is an issue of grave concern for the accuracy of the study.

Overall this is a very useful pilot study and I hope the larger study will shed light on some important questions in the trauma world.

VERSION 2 - AUTHOR RESPONSE

Response to Reviewer 1, Dr. Feroze Sidhwa, San Joaquin General Hospital:

Q1 Improvements in patient outcomes could take much longer than a few months to manifest, and may also be dependent on a certain level of training being achieved in the unit. These limitations should be addressed.

> R1 We agree, especially some outcomes may take substantially longer than the follow up of one month. We have added a note to the discussion section to address this.

Q2 “The principal investigator at each hospital selected the units for training.” How? Was this based on who conducts the trauma resuscitation?

> R2 Only units involved in the trauma resuscitation were eligible for training. The principal investigator selected the units based on their own mandate to involve the units in the study.

Q3 How do you know the accuracy of ICD-10 coding at these hospitals?

R3 The project officers conducting the ICD-10 coding have been trained using the WHO module on ICD-10 coding and their coding reviewed by the research team in previous projects.

Q4 Faculty not being included in the study because they are not typically directly involved in the initial management of trauma patients will be a major factor limiting generalizability of the findings. This cannot be helped and not every trial can be generalizable to the entire universe, but this should be noted in future conclusions and limitations.

R4 Good point. We have a highlighted this in the appropriate section in the discussion.

Q5 "We initially planned to use simple random sampling to select the units to be trained, but for pragmatic reasons this decision was left to the site principal investigator." Does this mean that the site principal investigator decided what kind of sampling to use, or that they decided on what units to be trained instead of using any kind of randomization?

R5 The principal investigator at each site decided what units to be trained instead of randomising these units. We have clarified this in the manuscript.

Q6 An "error in the data uploading process" that led to 1/10th of the data being collected in two out of seven clusters is very alarming. Isn't it possible this data uploading process error wasn't a source of random error? For example maybe it drastically reduced the data available from the two most incompetent clusters, regardless of training status? Unless this issue is addressed it would be a major source of potential error in a larger trial. This was mentioned briefly in the conclusion but I would encourage the authors to make clear that they understand that this is an issue of grave concern for the accuracy of the study.

R6 We agree that this is a major concern that cannot be allowed to happen in a full-scale trial. We have strengthened the language in the discussions section accordingly.

Q7 Overall this is a very useful pilot study and I hope the larger study will shed light on some important questions in the trauma world.

R7 Thank you very much and we agree.
